# Analysis of effect of thickness and surface treatment on sound transmission loss characteristics of natural fibres

**DOI:** 10.1038/s41598-025-14210-w

**Published:** 2025-08-07

**Authors:** Methil Vivek Shankar, N. H. Padmaraj, Sriharsha Hegde, G. M. Yash, Chandrakant R. Kini

**Affiliations:** Department of Aeronautical and Automobile Engineering, Manipal Institute of Technology, Manipal Academy of Higher Education, Manipal, 576104 Karnataka India

**Keywords:** Sustainable materials, Fibre treatment, Sound absorption, Sodium hydroxide, Natural fibres, Engineering, Materials science

## Abstract

Noise pollution, driven by rapid urbanisation and city expansion, has created a growing demand for innovative and bio-degradable sound absorption materials. Traditional solutions such as synthetic acoustic foams are widely used due to their efficiency and low cost but raise environmental concerns because of their non-biodegradable nature. This study explored the use of natural fibres—coir and sponge gourd—as environmentally friendly alternatives for sound insulation. The research focused on the effect of fibre thickness and surface treatment (using sodium hydroxide (NaOH)) on their acoustic performance. The Fourier Transform Infrared Spectroscopy (FTIR) technique was used to understand the effects of the treatment on the functional groups of the fibre. The surface modification of the fibre surface was studied using an optical microscope, Brumauer–Emmet–Teller (BET) analysis and by analysis of Scanning Electron Microscope (SEM) images. An impedance tube setup was used to measure the sound transmission loss for both the untreated and treated fibres. The results showed that increasing the thickness of both coir and sponge gourd fibres improves transmission loss at lower frequencies but reduces effectiveness at higher frequencies. Surface treatment had a material-dependent effect: sponge gourd fibres showed improved transmission loss due to increased surface roughness and airflow resistivity, whereas coir fibres exhibited a decline in performance after treatment. These findings contribute to a better understanding of how natural materials can be optimised for acoustic applications through structural modifications.

## Introduction

In the current world’s technological landscape, one of the rapidly emerging fields of research pertains to the management of sound, or in other words, sound absorption or insulation. This is particularly enforced or demanded due to the rise in noise pollution all around the world due to urbanisation and city-wide growth^1^. Thus, effective control measures are necessitated for the absorption or insulation of sound waves. The current standard, for example, utilised in automobiles, is the use of acoustic foams or panels due to their excellent sound absorption characteristics and low cost^2^. However, due to most of these types of panels utilising synthetic or chemical constituents in the basic construction, they are not particularly environmentally friendly or recyclable. Due to this, there is a growing need for natural fibre-based solutions that can be sustainable and cost-effective as well as readily available and that can perform the task of sound absorption while being environmentally friendly.

The research community has already displayed interest in the field of natural fibres and composites as a sound absorption device^3–6^. In a study conducted by Tiuc et al.^7^composite materials were developed using recycled particles of rubber, pine sawdust, and a binder made of polyurethane. The incorporation of these materials is aimed at addressing two key issues: waste disposal and noise pollution. The sound absorption capabilities of the composites were evaluated by calculating the absorption coefficient based on the Delany-Bazley model. Additionally, experimental tests were conducted using an impedance tube to verify the results obtained from the analytical model. The study concluded that the produced materials have the potential of being utilised in the manufacture of sound-absorption panels, which could be applied for soundproofing in multiple sectors, including industry, as well as in road, rail, and air transportation.

Dieckmann et al.^8^orchestrated a study examining the potential of non-woven feather fibres as a material for sound absorption in comparison to traditional oil-based synthetic fibres. In this research, waste feathers from poultry processing were cleaned, disinfected, and then converted into fibres. These fibres were processed into non-woven composite mats using commercial air-laying techniques. By manipulating the material composition and processing conditions, they were able to create samples with different thicknesses and densities. The sound absorption capabilities of the resulting materials were evaluated by utilising the impedance tube method. The findings suggested that non-woven feather mats could serve as a promising, eco-friendly alternative for sound insulation. For materials of the same thickness, their sound absorption performance was like that of conventional options, such as cellulose and mineral wool, as well as other natural fibre boards reported in prior research. However, the authors noted that additional studies are necessary to evaluate the environmental and social impacts of feather-based materials compared to traditional sound-absorbing options.

A study by Taban et al.^9^examined the acoustic capabilities of composites made from date palm fibres and polyvinyl alcohol. The research utilised an impedance tube method to assess the normal sound absorption coefficients of the materials. In addition, the sound absorption was determined through the Johnson-Champoux-Allard (JCA) model using a differential equation approach. Experimental results revealed that an increase in the thickness of the composites enhanced their sound absorption performance. Furthermore, the presence of an air gap behind the test samples inside the impedance tube was discovered to notably broaden the frequency range of sound absorption, particularly for thinner specimens.

In an experiment by Yan et al.^10^the acoustic properties of carbon fibre composite materials were determined using a standing wave (SW) tube setup. The researchers investigated how several factors affected the material’s sound absorption and transmission loss. Their findings revealed that the sandwich structure notably improved the sound absorption capabilities of the material. Furthermore, they found that an increase in the thickness of the composite material enhanced its overall acoustic performance. Similarly, Kumar et al.^11^conducted a study in which they measured the sound transmission loss (STL) and equivalent continuous sound pressure level of reinforced composites made from polypropylene/glass fibre. They compared these measurements with those of neat polypropylene and standard window glass. Their results offered insights into the material’s acoustical performance by showing differences between the composite and traditional materials. In a different study by Bahl et al.^12^the transmission loss (TL) of various materials was examined under aircraft noise conditions. The primary aim was to assess the sound absorption capabilities of glass, polypropylene, and glass fibre-reinforced composites. It was found that glass had a higher absorption rate than both neat polypropylene and the composites, particularly when different fibre orientations were evaluated. Additionally, composites with a 10% fibre volume fraction showed improved performance compared to the other tested materials. This enhancement was linked to the reduced porosity of the material as fibre content increased, which ultimately decreased sound transmission. In fact, the composite with the 10% fibre volume fraction exhibited STL comparable to that of glass.

A study by Gliscinska et al.^13^examined the sound absorption properties of a composite made by blending flax fibres with polylactide (PLA) fibres. The researchers assessed different configurations, including flat composites, composite/pre-pressed nonwoven systems, and profiled composites. The results showed that shaping the composite plate with either convex or concave profiles enhanced sound absorption. Additionally, the plate orientation relative to the incident sound waves was crucial in determining performance. For composites that incorporated a pre-pressed nonwoven layer, sound absorption was higher when the rigid composite layer faced the incoming sound waves. An increase in the number of nonwoven layers further improved absorption and expanded the range of effective frequency of maximum absorption to include lower frequencies. In a similar investigation by Abdi et al.^14^sound absorption was measured for a composite panel made from coffee silver skin (CSS) and chrome shave (CS). Using an impedance tube, the coefficient spectra of sound absorption of the composite panel was recorded at normal incidence. These experimental results were subsequently compared with the predictions from the JCA model, where input parameters like tortuosity, characteristic lengths, porosity, and resistivity to flow were considered. The findings suggested that the composite material is well-suited for use in acoustic panels, particularly for environments like meeting rooms, offices, and classrooms, where diverse acoustic needs exist.

In a study by Mawardi et al.^15^oil palm wood was explored as a promising material for sound absorption and thermal insulation. Panels were fabricated from oil palm wood using hot pressing, with variations in particle size and pressing time. The findings indicated that particle size significantly affected the properties of the binderless panels, while pressing time had a minimal effect. Larger particle sizes enhanced thermal and sound insulation properties but also led to lower density, reduced water resistance, and decreased flexural strength. Similarly, a study by Lashgari et al.^16^investigated panels made from glued wood chips, identifying them as effective materials for sound absorption. In a similar research, Suhaeri et al.^17^ explored composite panels made from Scirpus grossus fibres, derived from giant bulrush. These panels incorporated a perforated aluminium sheet to enhance sound absorption. It was determined that both the thickness of the Scirpus grossus panels and the hole size in the aluminium sheet influenced the sound absorption characteristics of the material.

The current study focused on understanding the effect of fibre treatment and thickness on the acoustic performance of the coir and sponge gourd samples. The fibres were treated with 6% NaOH solution. The effect of the chemical treatment on the fibre surface was analysed using FTIR analysis, BET analysis, SEM imaging and an optical microscope, and the acoustic performance of the untreated and treated samples were determined with an impedance tube setup.

## Materials and methods

### Natural fibre materials utilised

**Fig. 1:**
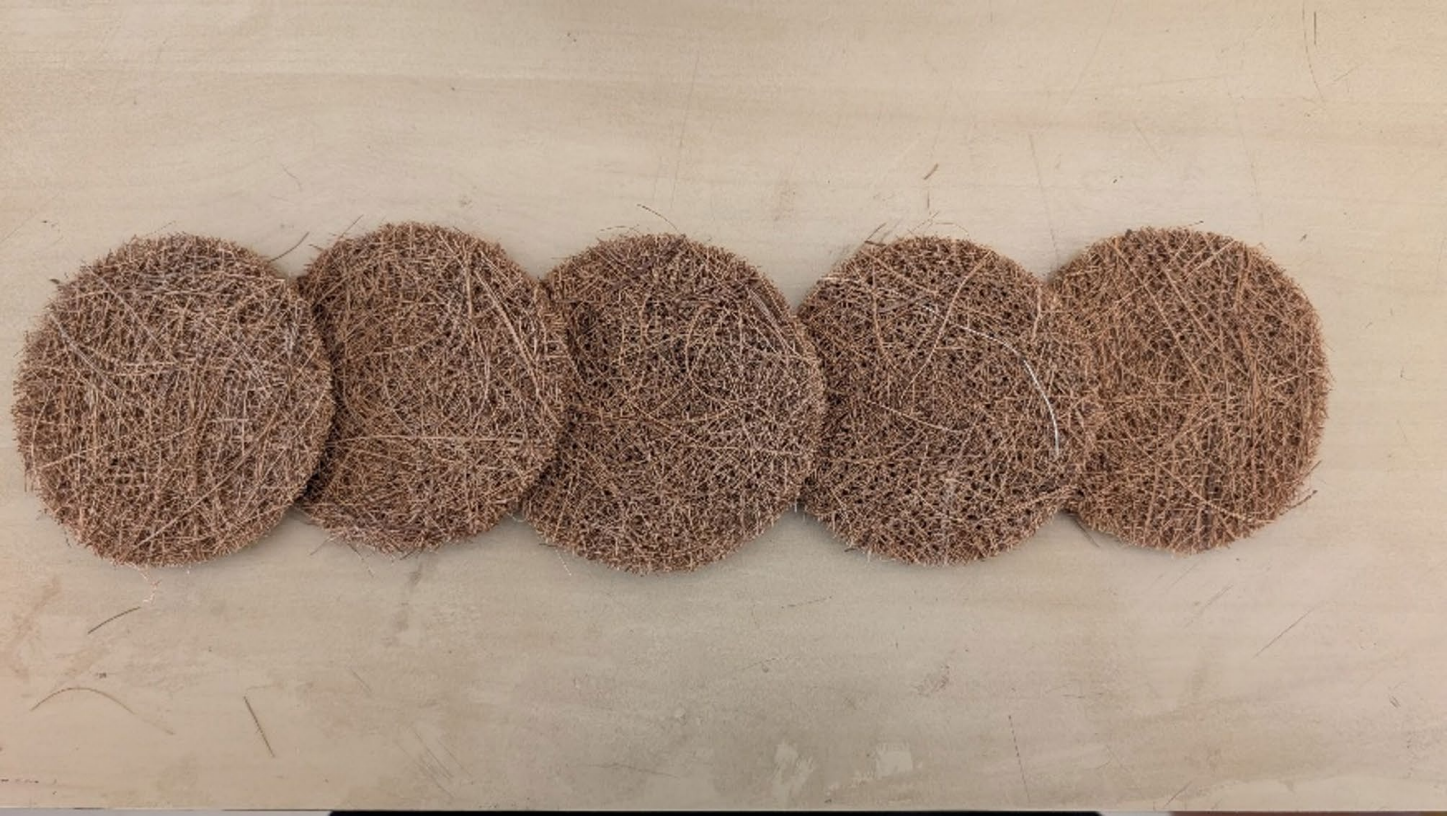
Coir sample for the acoustic test.

This study utilised two main natural fibre materials. The first is coir fibre, which was provided by Palmera Brushes, a company situated in Kanyakumari, India. The material was obtained in the form of round discs, as shown in Fig. 1.

**Fig. 2:**
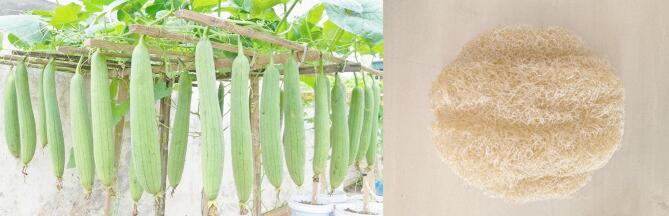
Sponge gourd fibre sample for the acoustic test.

The second material obtained was luffa gourd or sponge gourd fibres, which were provided by Mudhugiri Suvarna, a local company based in Tumakuru, India, as shown in Fig. 2.

The reason for the use of these two materials was due to their high availability and affordability, while also being easy to reproduce, making them a viable option for potential use as sound absorption material. Coir especially has already been widely adopted for multiple use cases, such as upholstery padding and mattresses, to even potentially being used as a reinforcement material for concrete composites^18^.

### Surface treatment of natural fibres

**Fig. 3:**
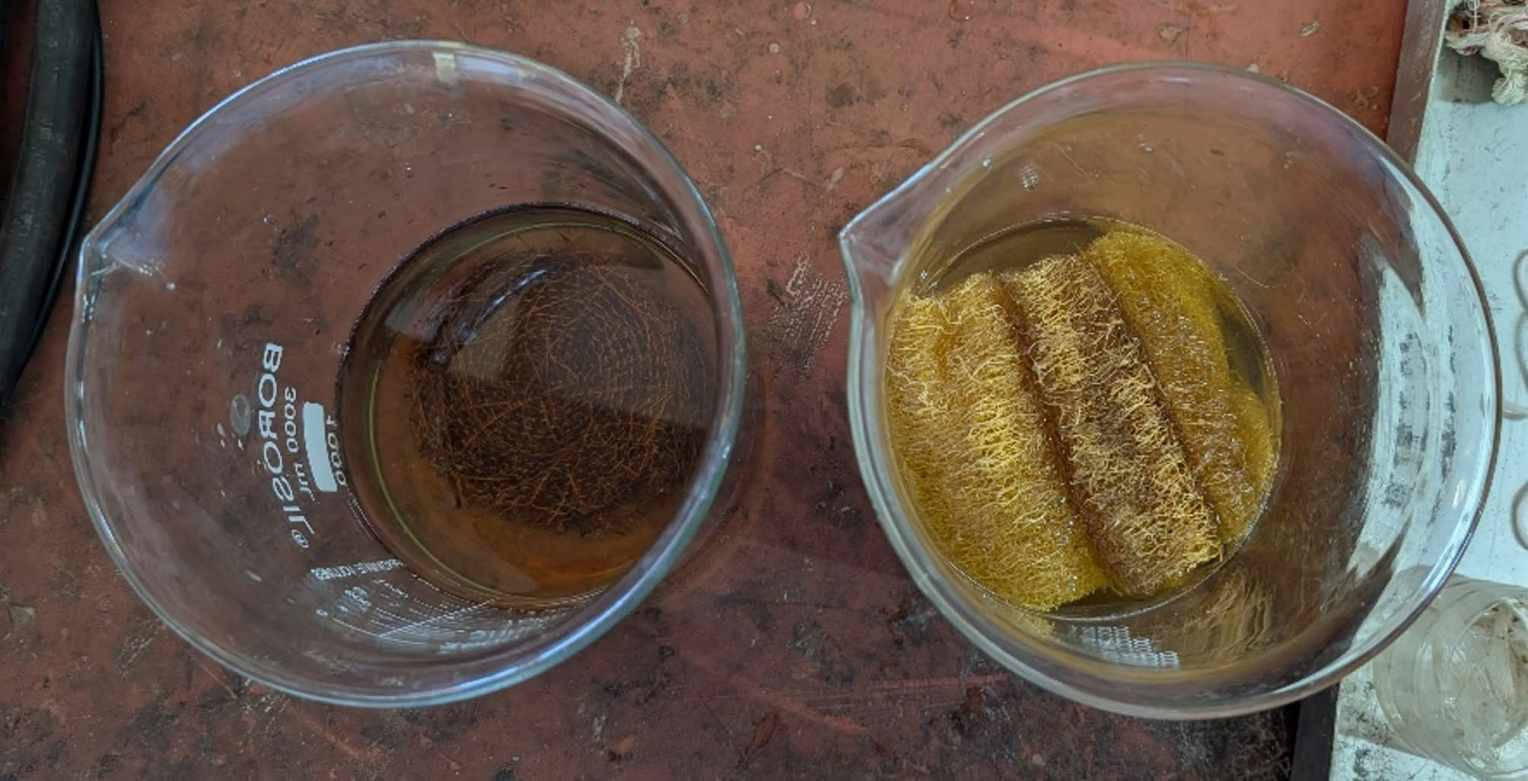
Surface treatment of fibre samples.

The procured natural fibres were subjected to a surface treatment process to improve the surface roughness of the fibre. From various potential surface treatment methods available, it was determined to make use of the alkaline treatment process for its simplicity and ease of obtaining materials^19,20^. The samples were immersed in a 6% concentration of sodium hydroxide (NaOH) solution for 24 h in ambient conditions, as shown in Fig. 3. The fibres were then removed and rinsed thoroughly with distilled water in order to remove the alkali solution from the fibre^21^. The boundary conditions for the treatment process were obtained from previous literature^22^. The cleaned samples were left to dry for a day in open air and then also heated in a hot-air oven for 3 h at 60 °C to remove any remaining moisture in the samples.

The fibre mats were then cut into diameters of 99 mm and 29 mm for performing the acoustic test using the impedance tube setup as per the ISO standard. The mats were also cut to ensure that they have a relative uniform thickness for all tested samples. The acoustic performance of the untreated and treated coir and sponge gourd fibres was studied by varying the thickness. The different types of samples used in the study and their nomenclatures are shown in Table 1.

**Table 1:** List of tested samples.

S. No.	Sample type	Nomenclature
1	Untreated Coir	UC
2	Treated Coir	TC
3	Untreated Sponge Gourd	USG
4	Treated Sponge Gourd	TSG
5	Double Layer Untreated Coir	2UC
6	Double Layer Treated Coir	2TC
7	Double Layer Untreated Sponge Gourd	2USG
8	Double Layer Treated Sponge Gourd	2TSG
9	Triple Layer Untreated Coir	3UC
10	Triple Layer Treated Coir	3TC
11	Triple Layer Untreated Sponge Gourd	3USG
12	Triple Layer Treated Sponge Gourd	3TSG

### FTIR analysis

The treated and untreated fibres were evaluated by the FTIR method to determine the modification of the functional groups of the fibres. Small, chopped pieces (average length of 2 mm) of the fibres in their treated and untreated forms were taken to perform FTIR analysis. The treated and untreated fibres were placed in a Bruker Alpha II FTIR spectrometer and scanned within a frequency range of 500 cm^− 1^ to 4000 cm^− 1^ in Attenuated Total Reflectance (ATR) mode.

### BET analysis

The treated and untreated fibres were evaluated by the BET method in order to determine the effect of the fibre treatment on the pore diameter of the fibres. Small pieces of the samples in powder form (average weight of 0.12 g) were placed in the sample holder of a BELSORP Mini X system, and the pore diameter and volume of the samples were determined using the BELMASTER software from the BET specific surface area.

### Surface morphology study

The study of the surface morphology of the treated and untreated fibres was conducted by first utilizing a Olympus BX53M optical microscope to obtain an image of the fibre’s surface at 10x magnification. Subsequently, a Zeiss EVO MA18 scanning electron microscope was utilized to obtain an image of the fibre’s structure at 400x magnification. The images received allowed for analysis of the changes of the fibre’s physical properties and surface morphology caused by the NaOH treatment.

### Acoustic testing

**Fig. 4:**
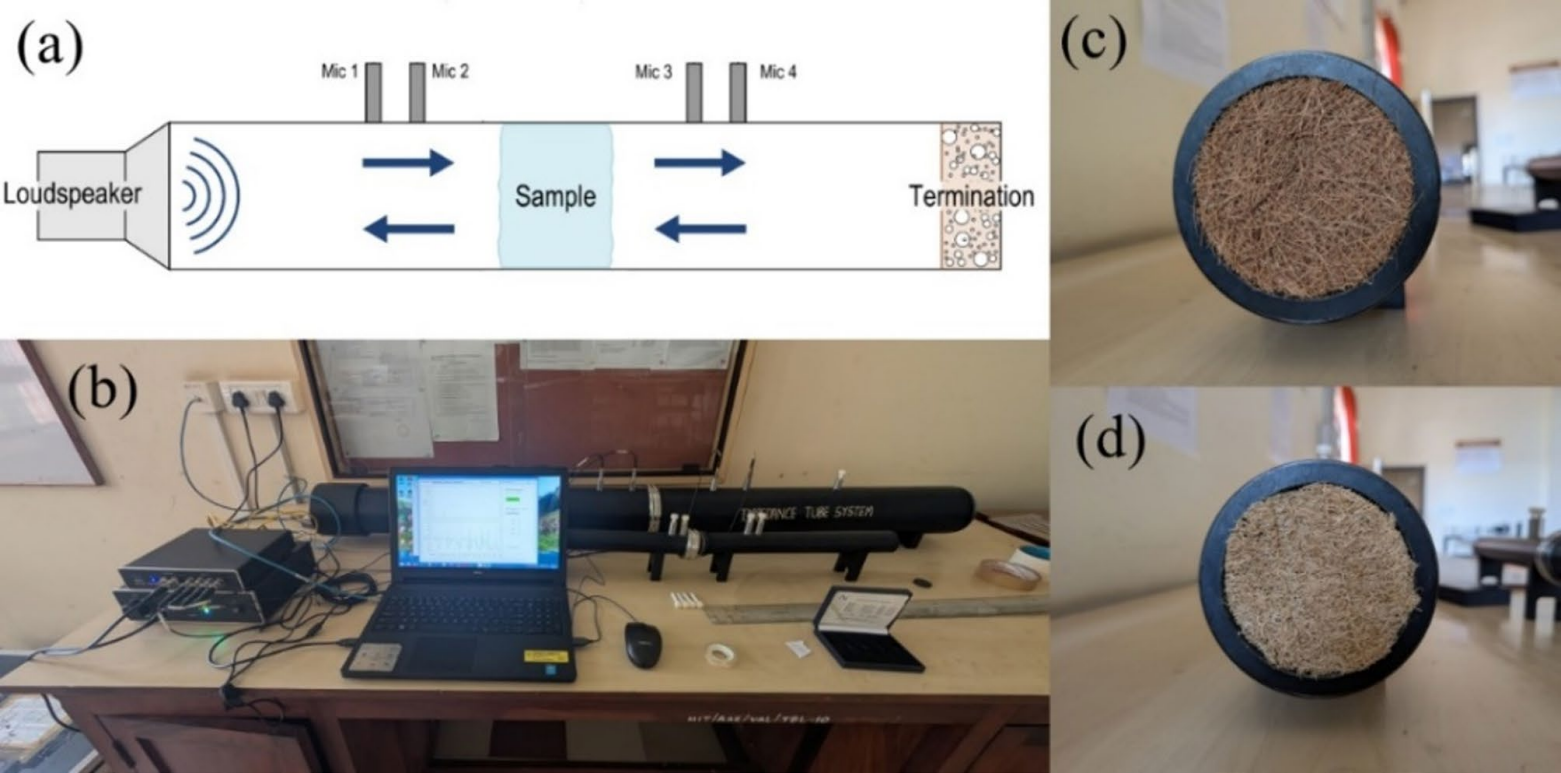
**a** Impedance tube setup schematic, **b** Experimental setup, **c** Coir sample placed in tube setup, **d** Sponge gourd sample placed in setup.

The samples were then tested for their transmission loss capabilities by utilising a BSWA SW impedance tube setup following the ISO 10534-2 standard^23^. The setup included a source tube (SW100-L) and an extension tube (SW100-E), with MPA416 microphones connected to an MC 3242 interface as shown in Fig. 4a. Figure 4b shows the experimental setup, and Figs. 4c and d show the 99 mm UC and USG samples placed in the larger tube.

The process of measuring transmission loss utilises a quad-microphone transfer function. The samples were placed one by one between the extension tube and the source tube. The specified measurement range on the tube (from 63 Hz to 6300 Hz) and the conditions of the environment, such as the atmospheric pressure, humidity and temperature, are entered into the interface system. Two microphone configurations, namely narrow and wide spacing, were employed. A loudspeaker, featuring a 400 mm diameter, 20 W power output, 8-ohm impedance, and frequency range of 20 Hz to 8000 Hz, was activated. After operating for around 20 min in each spacing, readings from the microphones were recorded across the specified frequency range of 63 Hz to 6300 Hz. The distribution of the sound transmission loss over the entire frequency range was then obtained^24^. Each fibre sample in varying thickness and in their treated and untreated forms was tested 3 times and the average of the results were recorded for analysis. The atmospheric conditions at the time of testing was at an average temperature of 35 °C with a humidity of around 60% while the pressure was set at a default of 101.325 kPa^25^.

## Results

### Effect of surface treatment on structure of Coir and sponge gourd fibres

The physical dimensions of the natural fibre samples were measured before and after undergoing surface treatment by utilising digital vernier callipers and a digital weighing scale with an accuracy of 0.001 g. The average thickness of the samples was measured at 10 different points. The densities of the materials were then calculated to help determine the effect of the surface treatment on the coir and sponge gourd fibres. The results of the physical measurements can be seen in Table 2.

**Table 2:** Physical properties of untreated and treated samples.

S. No.	Sample	Average Weight of sample (g)	Average Thickness of sample (mm)	Density of Sample (g/cm^3^)
1	Untreated Coir (UC)	4.60	5.33 ± 0.703	0.109
2	Untreated Sponge Gourd (USG)	3.52	5.13 ± 0.457	0.087
3	Treated Coir (TC)	3.81	5.33 ± 0.703	0.091
4	Treated Sponge Gourd (TSG)	2.25	5.13 ± 0.457	0.056

**Fig. 5:**
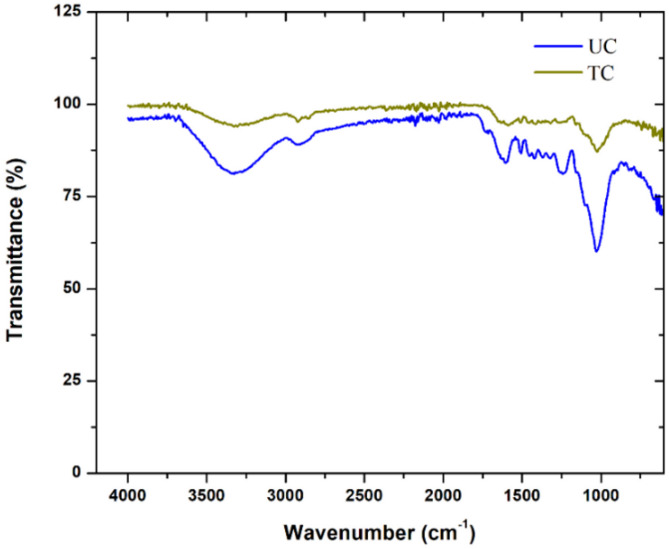
FTIR results of untreated and treated coir fibres.

From calculating the density of the fibres before and after the surface treatment, it was observed that the density of the coir sample reduced by nearly 20%, while for sponge gourd, the reduction in density after surface treatment was nearly 40%. The effect of fibre treatment on functional groups of coir and sponge gourd fibres was studied using the FTIR technique. The FTIR spectra of the untreated and treated coir samples are shown in Fig. 5.

**Fig. 6:**
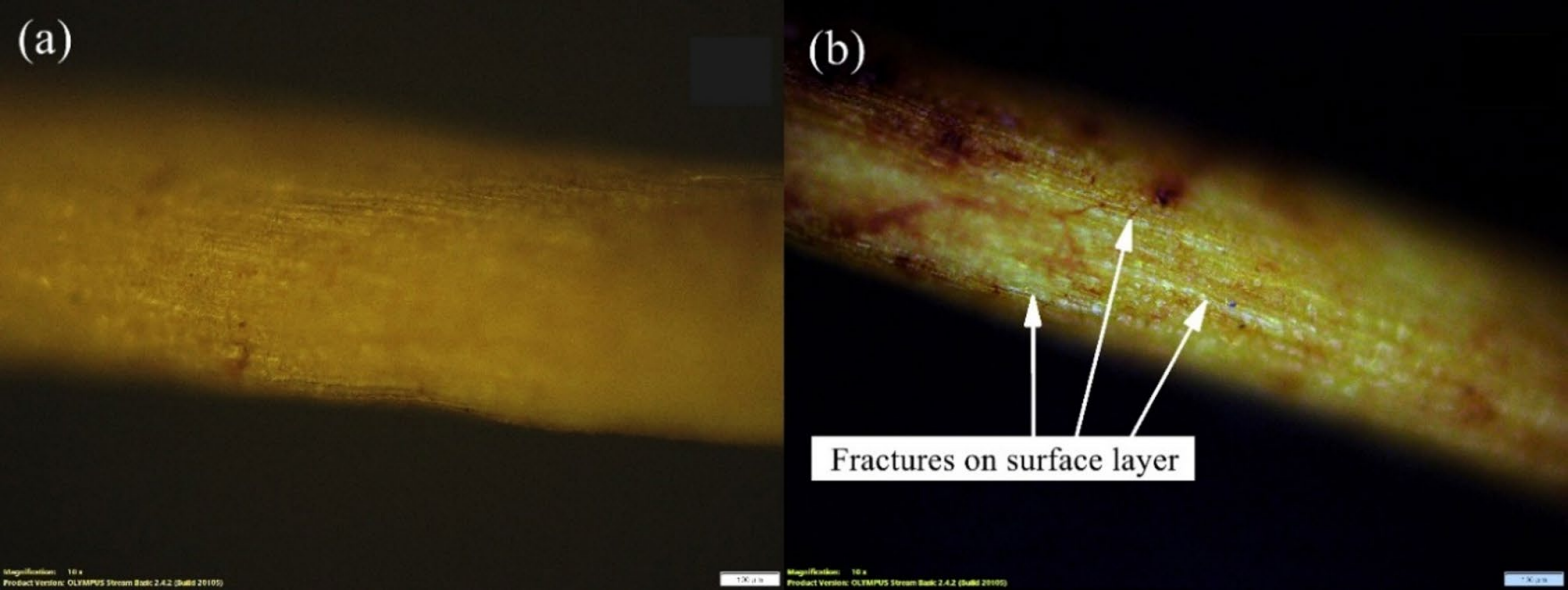
10x magnified image of **a** Untreated coir fibre and **b** Treated coir fibre.

In Fig. 5, it was observed that at around 3300 cm^− 1^, the UC sample experienced a peak at around 80% transmittance due to the vibration of the stretching hydroxyl (-OH) groups in the arrangement of the fibres, the reduction of this peak to around 95% transmittance in the TC sample indicated the removal of around 15% of the hydroxyl group from the surface of the fibre. Similar conclusions were also obtained from the increase in transmittance of around 90–95% at a wave number of 2900 cm^− 1^, signifying a 5% reduction of -CH bonds. The transmittance increases at 1600 cm^− 1^ from 85 to 95% and at 1000 cm^− 1^ from 60 to 90% which also signified a 10% reduction in -CH_2_ bonds and a 30% reduction in skeletal C-O bonds from the composition of the surface of the fibre. This can be observed in Fig. 6, which shows the fibre surface of the untreated and treated fibres obtained using the optical microscope. The microscopic analysis of the treated coir fibre (Fig. 6(b)) showed the formation of multiple surface cracks compared to the untreated fibre surface (Fig. 6(a)). The fracturing of the surface layer was due to the removal of the hemicellulose and lignin layers from the fibre surface. This would result in a decrease in the fibre’s overall diameter and an increase in the surface roughness. The removal of these specific bonds also would result in a reduction of the fibre’s overall rigidity, making it more flexible.

**Fig. 7:**
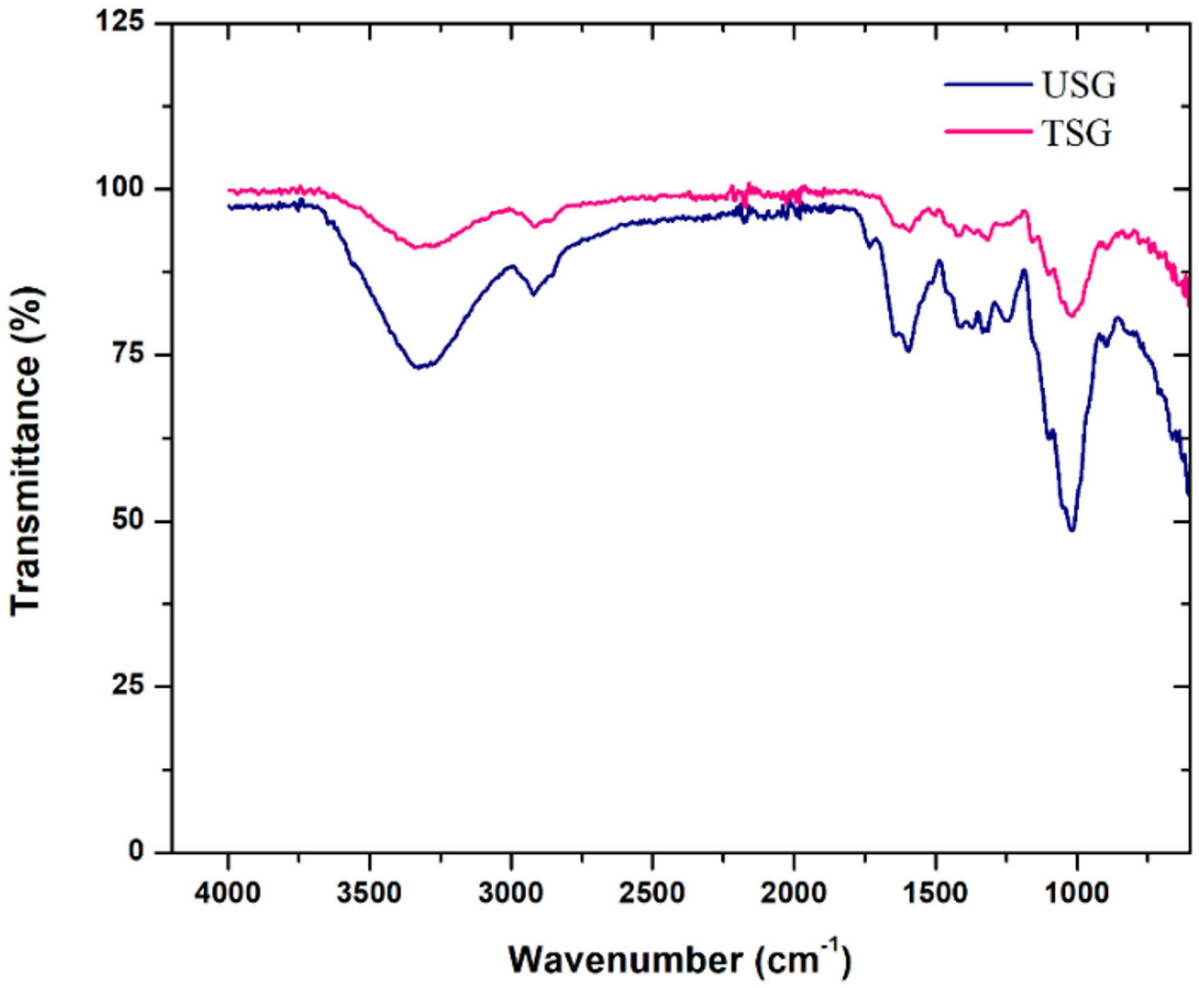
FTIR results of untreated and treated sponge gourd fibres.

**Fig. 8:**
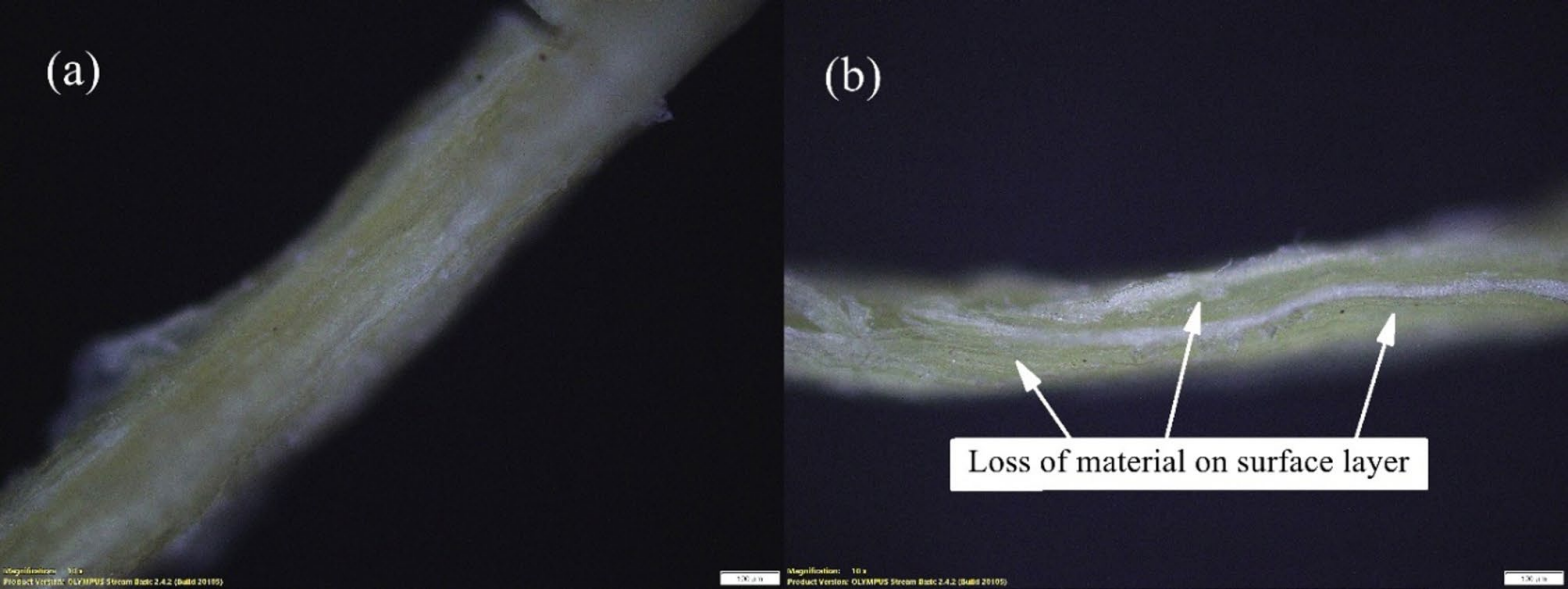
10x magnified image of **a** Untreated sponge gourd fibre and **b** Treated sponge gourd fibre.

Figure 7 shows the FTIR spectra of treated and untreated sponge gourd fibres. The spectra showed an increase in the transmittance at around 3300 cm^− 1^ from around 75% for the USG sample to 90% for the alkaline-treated TSG sample, indicating the removal of around 15% of the hydroxyl group from the surface of the fibre. The increased transmittance at 2900 cm^− 1^ from 85 to 95% also indicated a reduction of 10% in -CH bonds. Another variation of TSG compared to USG is the increase in transmittance at 1600 cm^− 1^ from 75 to 95% and at 1000 cm^− 1^ from around 50–80%, which signified a 20% reduction in -CH_2_ bonds and a 30% reduction in skeletal C-O bonds from the composition of the surface of the fibre. Like the coir, this also indicated the removal of the layers of lignin and hemicellulose from the fibre surface, as shown in Fig. 8a and b. The microscopic analysis of the treated sponge gourd fibre (Fig. 8b) showed the removal of material from the fibre surface compared to the untreated fibre surface (Fig. 8(a)) and similarly to the treatment of coir, this would have resulted in a reduction in the fibre’s rigidity. Compared to the treatment of the coir fibre, the surface treatment of the sponge gourd fibre was observed to be more effective on the sponge gourd fibre, as observed by the magnitude of the reduction of the functional groups. Thus, the decrease in diameter and increase in roughness of the fibres were considered to be much more significant compared to the reduction experienced in the coir. This was further corroborated by the reduction in density observed in Table 2.

**Fig. 9:**
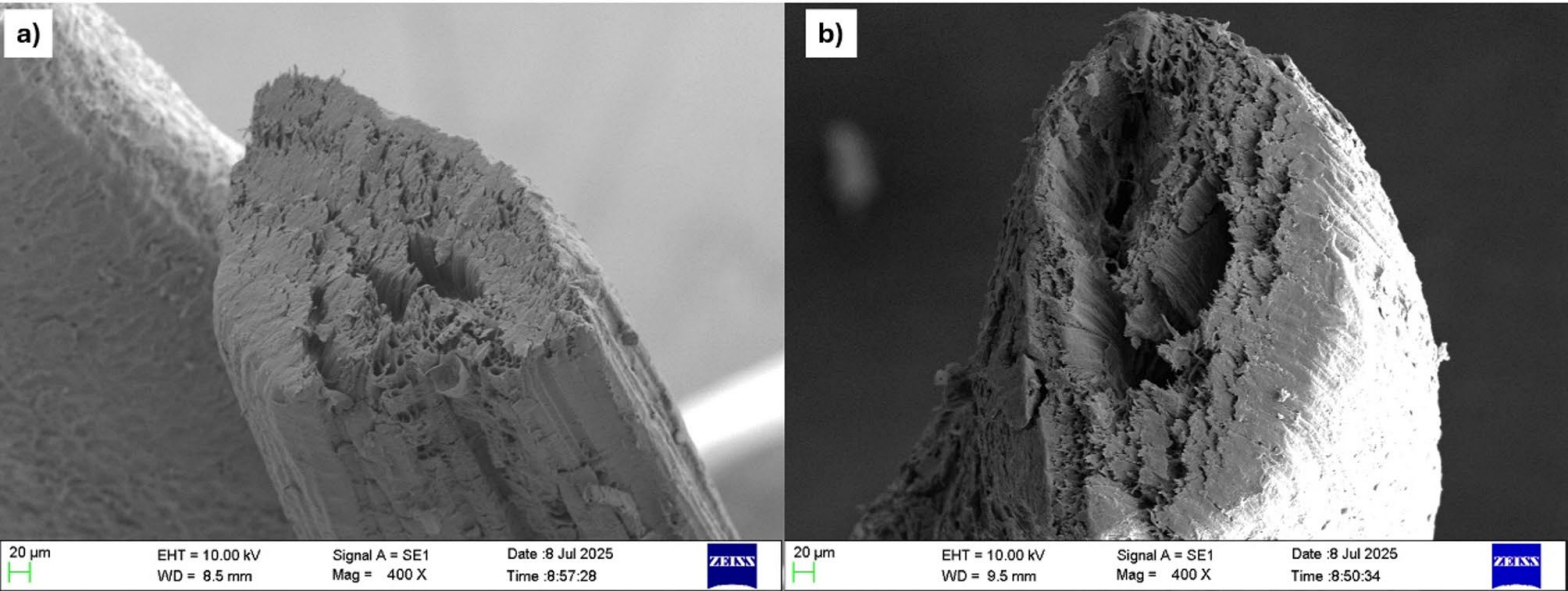
SEM images of **a** Untreated coir fibre and **b** Treated coir fibre.

Figure 9(a) and 9(b) shows the SEM images of untreated and treated coir fibre obtained by SEM. The analysis of the SEM image of the treated coir fibre in Fig. 9(b) showed that the fibre experienced an increase in porosity. SEM images also revealed the separation in the microfibril structure post alkali treatment due to the removal of the lignin and hemicellulose layers.

**Fig. 10:**
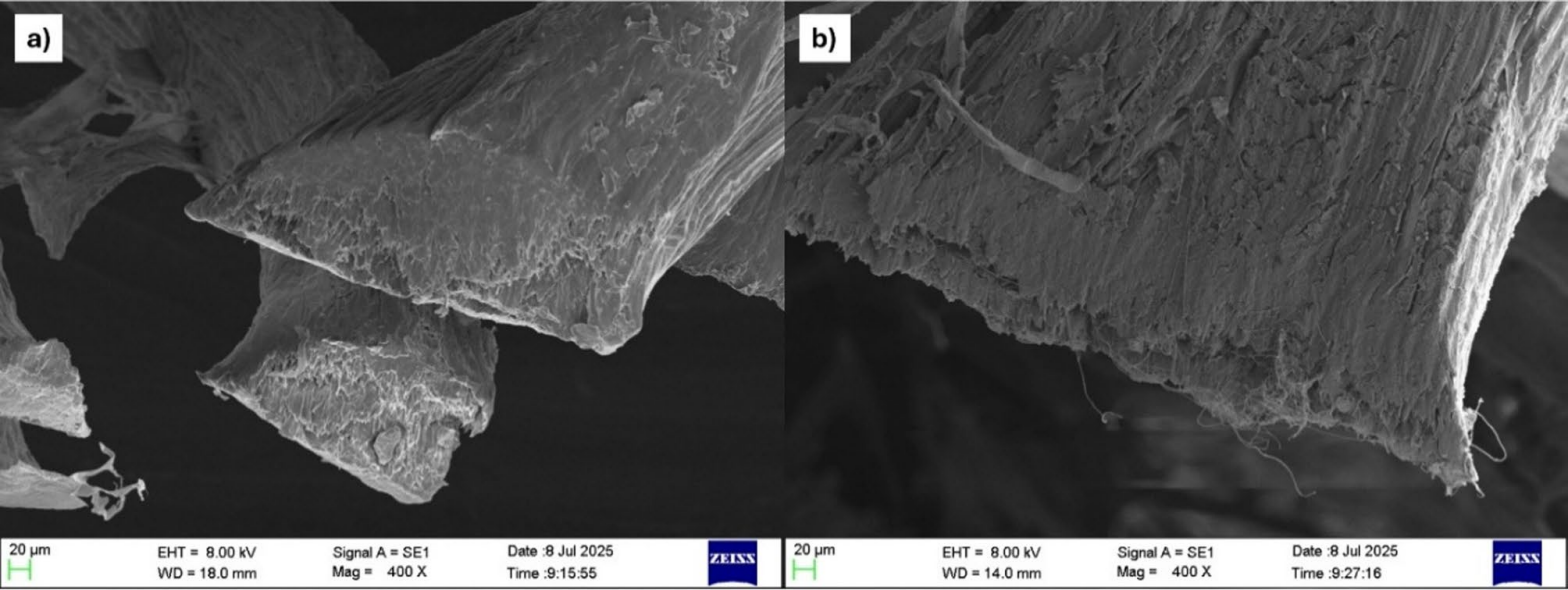
SEM images of **a** untreated sponge gourd fibre and **b** treated sponge gourd fibre.

Figure 10a and b displays the SEM imaging of untreated and treated sponge gourd fibre respectively. Compared to the untreated fibre sample in Fig. 10a, the analysis of the SEM image of the treated sample in Fig. 10b also showed an increase in porosity due to the removal of the hemicellulose and lignin layers from the fibre surface due to the alkali treatment.

**Fig. 11:**
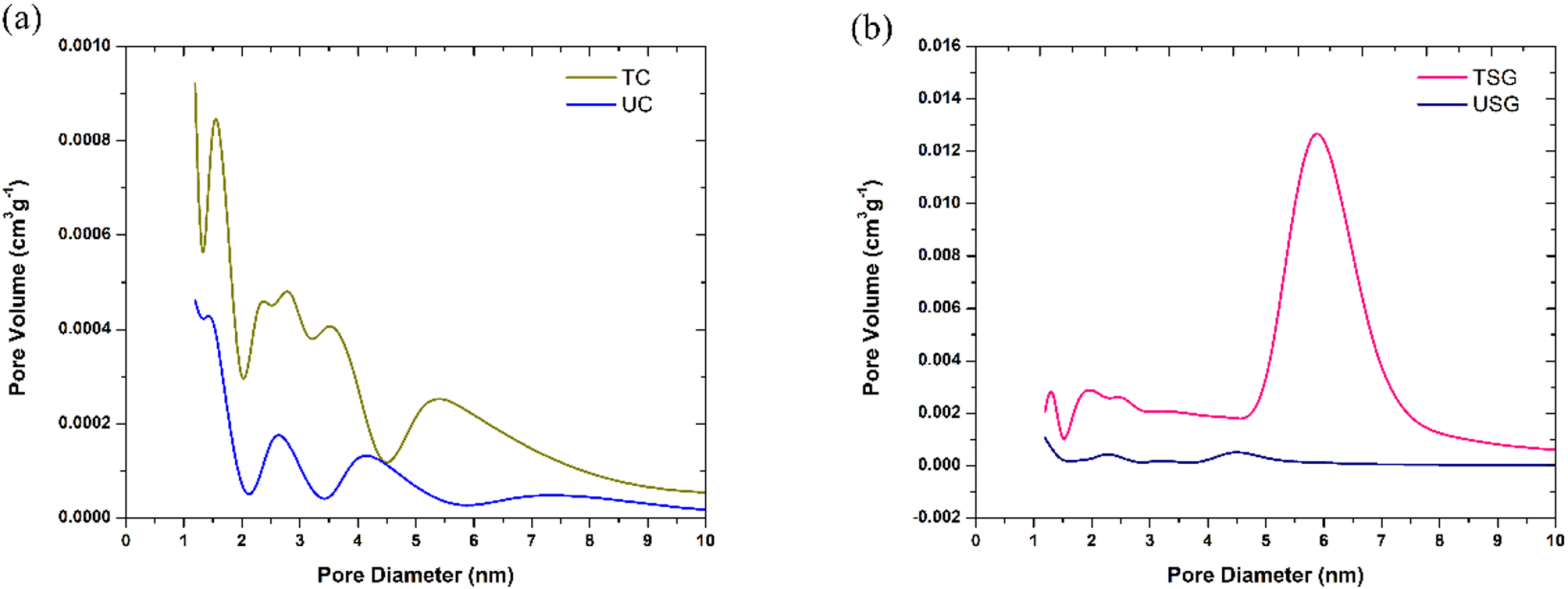
Comparison of BET Analysis results between **a** UC and TC, **b** USG and TSG.

The results of the BET analysis of the tested fibre materials are shown in Fig. 11. From Fig. 11(a) and 11(b), the measurements for the surface volume, area and pore diameter were achieved for the untreated and treated forms of the tested fibres. These values can be observed in Table 3.

**Table 3:** BET analysis Results.

S. No.	Sample	Surface volume (cm^3^/g)	Surface area (m^2^/g)	Pore diameter (nm)
1	UC	0.0141	0.223	2.53
2	TC	0.0413	0.259	4.61
3	USG	0.0531	0.231	4.19
4	TSG	0.0619	0.269	6.31

From Table 3, it was observed that the alkaline treatment caused an increase in the surface volume and in the pore diameter of the coir and sponge gourd fibre. As for the surface area, while the sponge gourd experienced around a 17% increase from 0.231 m^2^/g to 0.2695 m^2^/g, coir experienced an increment of around 14% in surface area from 0.223 m^2^/g to 0.249 m^2^/g. The treatment of coir with alkali solution also resulted in the formation of pores in the range of 5 to 6 nm.

### Effect of surface treatment on natural fibre transmission loss behaviour

**Fig. 12:**
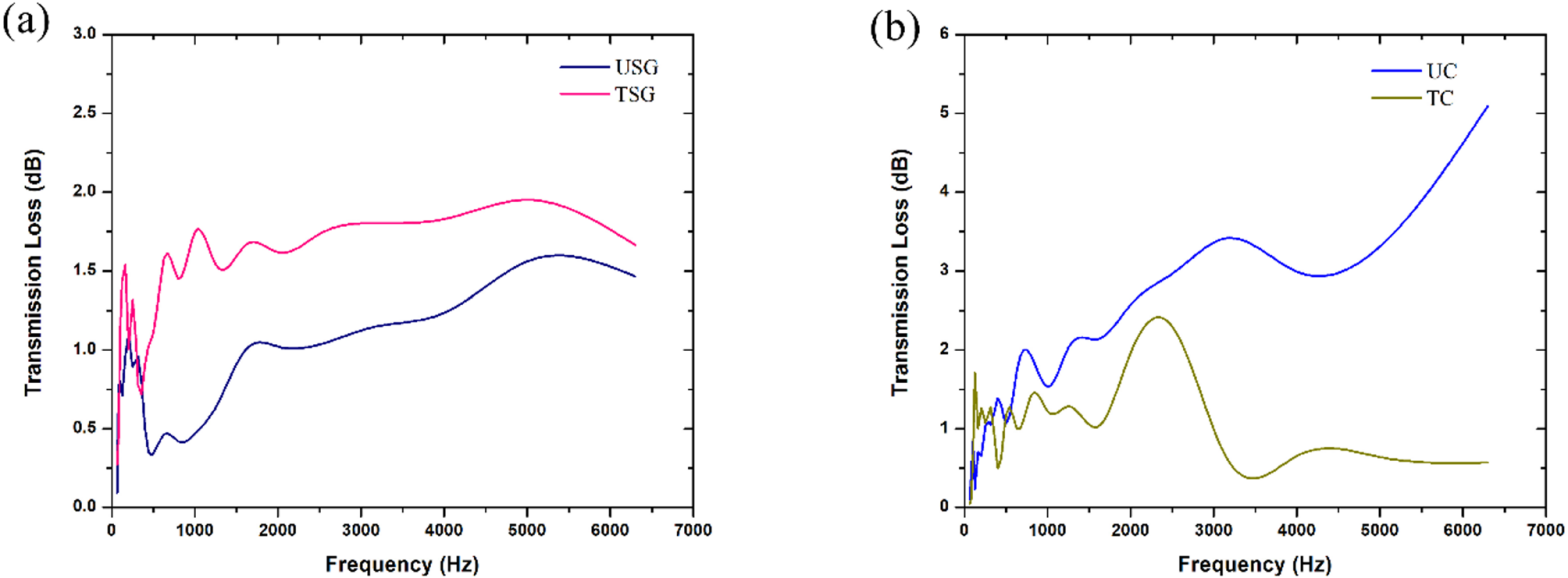
Comparison of transmission loss behaviour between **a** USG and TSG, **b** UC and TC.

Figures 12(a) and 12(b) shows the comparison of the results of the transmission loss test between USG and UC against their treated equivalents.

The analysis of the acoustic behaviours showed that the TSG sample performed better than the USG sample for most of the frequency range, but the rate of improvement started to decline early on from beyond 1600 Hz, with both the samples only able to absorb 1.5 dB in sound at the end of the frequency range. However, the TSG sample attained a maximum peak of nearly 2 dB at 5000 Hz, while the USG sample peaked at only 1.6 dB at the same frequency.

Figure 12b shows that unlike sponge gourd, the overall transmission loss capabilities of the coir material reduced after the surface treatment, with the TC sample performing at a reduced capacity compared to the UC sample for most of the frequency range. The TC sample only achieved a peak of 2.3 dB at 2500 Hz before reducing to 0.6 dB for the rest of the range from 3150 Hz onwards, while the transmission loss of the UC sample continued to increase as the frequency increases.

**Fig. 13:**
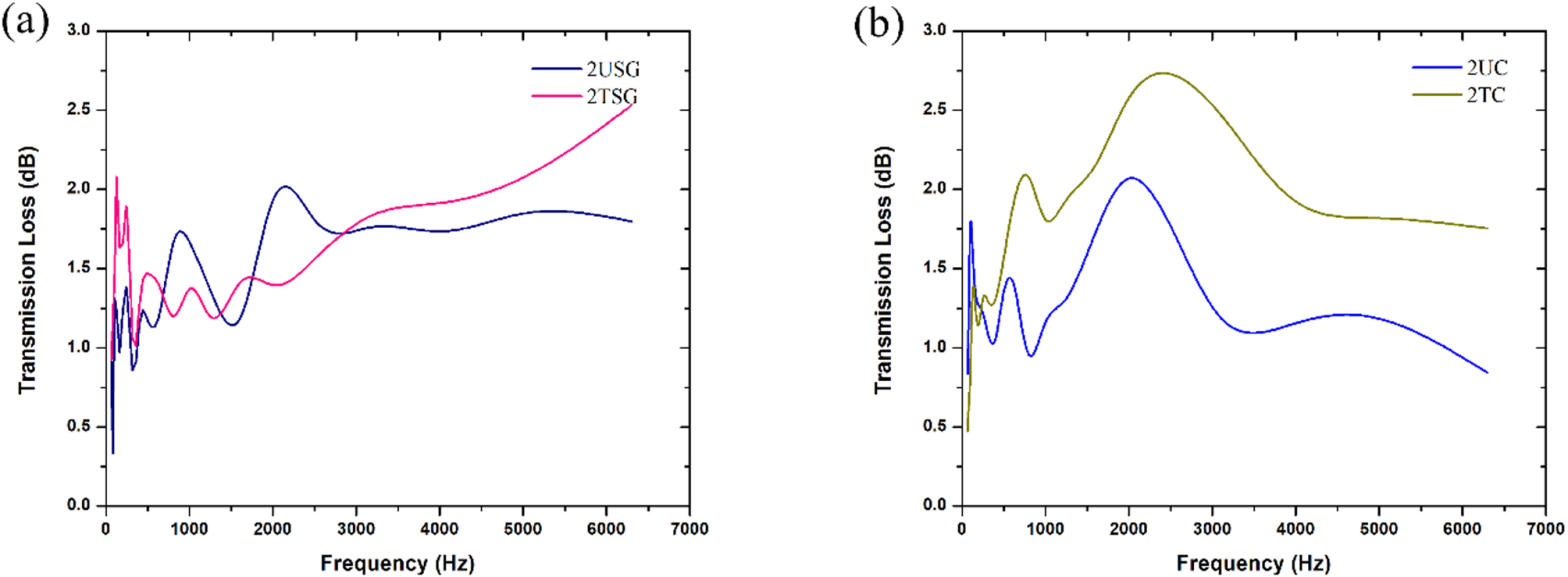
Comparison of transmission loss behaviour between **a** 2USG and 2TSG, **b** 2UC and 2TC.

Figure 13a shows that the 2TSG sample (thickness of 10.26 mm) continued to perform better than the 2USG sample (thickness of 10.26 mm), which only performed higher at certain frequencies like 1000 Hz, where the 2USG achieved a peak of 1.7 dB compared to the 2TSG, which absorbed only 1.4 dB in sound. Another exception is at 2000 Hz, where the 2USG peaked at 1.9 dB compared to the 2TSG, which absorbed only 1.4 dB. However, beyond 3000 Hz, the transmission loss of the 2TSG continued to rise while the 2USG stagnated, absorbing only around 1.8 dB for the higher frequencies.

For the coir samples, Fig. 13b shows that, unlike the comparison made at single-layer thickness, 2TC (thickness of 10.66 mm) performed better than 2UC (thickness of 10.66 mm), with 2TC absorbing a maximum of 2.8 dB at 2500 Hz, while 2UC achieved a maximum of only 2.2 dB at 2000 Hz. Another observation made from the analysis was that for 2TC, the performance of the sample stagnated beyond 2500 Hz, with the sample only absorbing around 1.8 dB at the higher frequencies. Similarly, 2UC also stagnated in its performance beyond 2000 Hz, with the fibre absorbing only around 1.2 dB at higher frequencies.

**Fig. 14:**
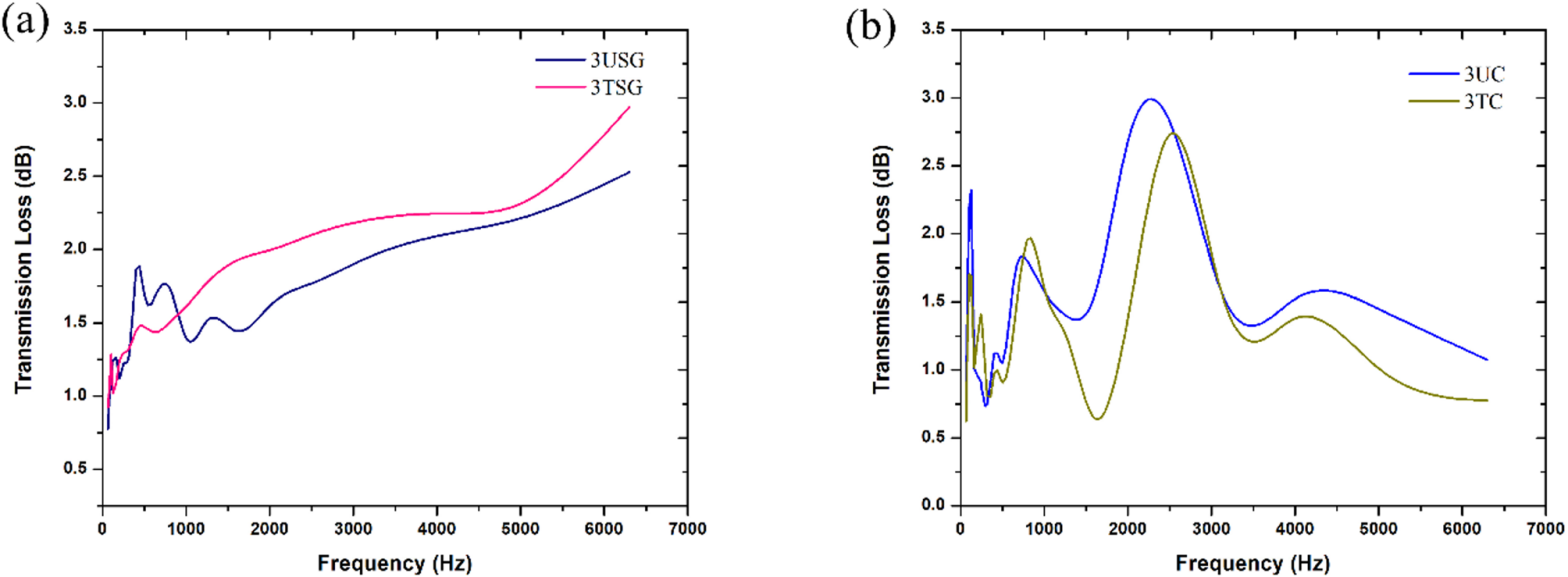
Comparison of transmission loss behaviour between **a** 3USG and 3TSG, **b** 3UC and 3TC.

Figure 14a shows that beyond 1000 Hz, the 3TSG sample (thickness of 15.39 mm) continued to absorb acoustic waves more effectively than the 3USG sample (thickness of 15.39 mm). At lower frequencies from 400 to 1000 Hz, it was observed that 3USG performed slightly better, with the sample absorbing an average of 1.8 dB, while 3TSG absorbed an average of only 1.5 dB in the same frequency range. Beyond 1000 Hz, as the frequency increased, both 3USG and 3TSG continued to improve their ability to absorb sound, with 3TSG absorbing around 0.3 dB of sound higher than the 3USG for the remainder of the frequency range.

From Fig. 14b, it was observed that the 3TC sample (thickness of 15.99 mm) performed lower than the 3UC (thickness of 15.99 mm), with the 3TC sample only able to absorb 2.7 dB of sound at 2500 Hz. The 3UC sample was able to perform slightly higher with it absorbing 2.8 dB of sound at the same frequency. Both 3UC and 3TC experienced a reduction in their ability to absorb sound beyond the 3000 Hz frequency, with 3UC only absorbing around 1.5 dB in the frequency range and 3TC only absorbing around 1.2 dB.

### Effect of increasing thickness on transmission loss behaviour of natural fibres

Figures 12b, 13b and 14b showed that at the lower frequencies, up to 2500 Hz, all three untreated coir samples displayed increasing levels of transmission loss as the frequency increases. However, beyond 2500 Hz, the transmission loss capabilities of the 2UC and 3UC samples decreased significantly to around 1-1.5 dB for the rest of the frequency range. This was in sharp contrast to the UC sample, which continued to increase its rate of transmission loss as the frequency increased. Figures 12b, 13b and 14b also show that just like the untreated samples, the increase in transmission loss capabilities of the treated coir appeared to rise with the increase in thickness, especially at lower frequencies up to around 3150 Hz, with the rate of improvement then declining and then plateauing for the remainder of the frequency range, with the 2TC sample performing the best by absorbing a maximum of 2.7 dB at 2000 Hz, followed by the TC sample absorbing 2 dB and the 3TC sample absorbing 1.4 dB at the same frequency.

For the untreated sponge gourd samples, Figs. 12a, 13a and 14a show that for the entirety of the frequency range, the 3USG sample was able to absorb higher amounts of sound energy from the incident acoustic waves, followed by the 2USG sample and then by the USG sample. The behaviour was repeated after alkaline treatment, with the 3TSG sample having the higher transmission loss, followed by the 2TSG sample and the TSG sample. Both the 2TSG and TSG samples performed very similarly up to 5000 Hz, with the TSG sample peaking while the 2TSG sample continued to increase its transmission loss as the frequency increased.

## Discussion

From the results obtained, it can be verified that increasing the thickness of natural fibres can improve transmission loss capabilities of the material at low or, in some cases, medium frequencies. However, at high frequencies, the rate of improvement can begin to decline or may even perform worse than a single layer of the same material. A potential cause for the ability of single-layer sample to absorb sound increasingly with the increase in frequency is due to the increasing level of activation of the fibre. The vibrating air between the fibres in the sample accelerates the interaction between the pore’s edges and the air. This results in a viscous force being created which converts the sound energy into heat, thus allowing for better transmission loss capabilities as the frequency increases. In the case of the double-layer and triple-layer samples, the increase in transmission loss in the lower frequencies could be attributed to the inner layer’s ability to absorb acoustic waves at low frequencies. But, at higher frequencies, the acoustic waves are largely absorbed by the surface of the samples and do not reach the inner layer, thus causing the transmission loss to lower at higher frequencies. This corroborates with previous studies. In a study conducted by Qui and Enhui^26^the effect of an increase in thickness on the transmission loss of wool fibreboard was analysed. The study found that the transmission loss increased at lower frequencies but appeared to fluctuate at higher frequencies. However, the study also found transmission loss to improve at higher frequencies with increasing density. In another study by Gómez Escobar et al.^27^the effect of density and thickness on the transmission loss was analysed for acoustic panels made from used cigarette butts. The study concluded that an increase in thickness or density resulted in the shifting of the absorption capabilities of the sample towards lower frequencies. The results obtained from the untreated and treated samples corroborate the previous studies.

The FTIR analysis and analysis of the magnified images of the treated and untreated natural fibre samples also show that the alkaline treatment resulted in the removal of the layer of fibres containing hemicellulose and lignin from the surface of the samples, with it proving to be more effective for the sponge gourd fibre compared to the coir sample. The removal of these bonds resulted in an increase in the surface roughness and a potential decrease in the fibre diameter. In a previous study conducted by Nasidi et al.^22^NaOH treatment was conducted on kenaf fibres in varying levels of concentration, and it was found from FTIR analysis that after the treatment process, the fibres had lost the surface layer of fibres containing hemicellulose and lignin, similar to the current study made.

The effect of surface treatment on the transmission loss capabilities of the natural fibres appeared to differ depending on the material. For coir, the treated sample displayed worse transmission loss capabilities compared to the untreated sample for the entirety of the frequency range, highlighting the need for further studies to understand the effect surface treatment has on the material. However, for sponge gourd, the performance was found to improve at all three levels of thickness. A potential cause for the improved performance in the treated sample could be attributed to the decrease in the diameter of the fibre and increase in surface roughness caused by the treatment itself. The increase in surface roughness and the decrease in diameter of the fibres can be attributed to the loss of specific chemical bonds on the fibre surface, as identified in the FTIR analysis. The removal of the hemicellulose and lignin bonds on the fibre surface also result in reduction in the fibre’s rigidity. Due to the increase in surface roughness and reduction in diameter, there was an increase in the air flow resistivity leading to better transmission loss of sound waves. This effect can be linked to viscous boundary layer theory, where the rougher surface provides a larger area for viscous energy dissipation, leading to improved transmission loss^28,29^. Another potential cause of the increase in transmission loss is the increased friction between the fibre and the sound waves restricting their movement through the treated sample. The reduction in rigidity also allows for the fibres to be more flexible, allowing for improvements in the fibre’s damping capabilities, especially at the low and medium frequency ranges. In the study by Nasidi et al.^22^it was also found that surface treatment improved the transmission loss capabilities of the kenaf fibres, with the sample treated with 6% NaOH concentration performing the best, especially at lower frequencies, thereby correlating with the results obtained with the sponge gourd fibre.

From the BET analysis, it was observed that the sponge gourd and coir experienced an increase in pore diameter as well as in increase in surface volume and surface area post alkaline treatment. This led to an increase in the fibre porosity. which could be correlated to the reduction in fibre diameter as well as increase in the surface roughness. This was further highlighted in the SEM analysis where the treated fibres displayed increased porosity. The analysis of the SEM images of the treated coir fibre detected a potential collapse of the fibrillar structure. This can be considered as a potential cause of the reduction in the fibre’s ability to absorb sound waves post alkaline treatment. In a review by Mamtaz et al.^30^it was determined that an collapse in a natural fibre’s fibrillar structure could result in a reduction in the fibre’s ability to absorb sound waves due to the reduced interaction between the fibre surface and the sound waves.

Future studies can be focused on better understanding the effects of thickness, density or even different surface treatment techniques can have on the ability of natural fibres to absorb acoustic waves. This can be achieved by additional testing by micro-CT, acoustic impedance modelling or by FESEM or Field Emission Scanning Electron Microscope. Further studies can also emerge to determine how the different materials may perform in various combinations with each other.

## Conclusions

The effect of varying thickness and surface treatment on the transmission loss capabilities of the coir and sponge gourd fibre was analysed using the impedance tube setup. The study utilised NaOH treatment to modify the surface morphology of the fibre. The effect of surface treatment was confirmed by FTIR, BET analysis as well as by observing for any changes in the structure by optical microscope and SEM. The study found that increasing the thickness of coir or sponge gourd fibres resulted in an increase in the transmission loss at lower frequencies but would decrease at higher frequencies. This behaviour was also repeated by the treated fibre samples when their respective thicknesses were also increased. The effect of surface treatment on the samples differed between the two materials. The transmission loss of the sponge gourd fibre increased after undergoing surface treatment due to the increase in surface roughness and airflow resistivity. However, the transmission loss capabilities of the coir sample reduced after undergoing surface treatment potentially due to the collapse of the fibrillar structure post alkali treatment. The results and observations made can assist in future works and studies that make use of combinations of multiple natural fibres as well as composite panels utilizing bio-resins.

## Data Availability

Data sets generated during the current study are available from the corresponding author upon reasonable request.
